# Regulation of the brain isoprenoids farnesyl- and geranylgeranylpyrophosphate is altered in male Alzheimer patients

**DOI:** 10.1016/j.nbd.2009.05.005

**Published:** 2009-08

**Authors:** Gunter P. Eckert, Gero P. Hooff, Dana M. Strandjord, Urule Igbavboa, Dietrich A. Volmer, Walter E. Müller, W. Gibson Wood

**Affiliations:** a Department of Pharmacology, Biocenter Niederursel, University of Frankfurt, Campus Riedberg, Max-von-Laue-St. 9, 60438 Frankfurt, Germany; b Department of Pharmacology, School of Medicine, University of Minnesota, Geriatric Research, Education and Clinical Center, VAMC, Minneapolis, MN 55417, USA; c Medical Research Council, Human Nutrition Research, Elsie Widdowson Laboratory, Fulbourn Road, Cambridge CB1 9NL, UK

**Keywords:** Alzheimer disease, HMG-CoA reductase, Isoprenoids, Farnesylpyrophosphate, Geranylgeranylpyrophosphate, Prenylation, GTPases, Cholesterol, Statins

## Abstract

Post-translational modification of small GTPases by farnesyl- (FPP) and geranylgeranylpyrophosphate (GGPP) has generated much attention due to their potential contribution to cancer, cardiovascular and neurodegenerative diseases. Prenylated proteins have been identified in numerous cell functions and elevated levels of FPP and GGPP have been previously proposed to occur in Alzheimer disease (AD) but have never been quantified. In the present study, we determined if the mevalonate derived compounds FPP and GGPP are increased in brain grey and white matter of male AD patients as compared with control samples. This study demonstrates for the first time that FPP and GGPP levels are significantly elevated in human AD grey and white matter but not cholesterol, indicating a potentially disease-specific targeting of isoprenoid regulation independent of HMG-CoA-reductase. Further suggesting a selective disruption of FPP and GGPP homeostasis in AD, we show that inhibition of HMG-CoA reductase *in vivo* significantly reduced FPP, GGPP and cholesterol abundance in mice with the largest effect on the isoprenoids. A tentative conclusion is that if indeed regulation of FPP and GGPP is altered in AD brain such changes may stimulate protein prenylation and contribute to AD neuropathophysiology.

## Introduction

Post-translational modification of proteins with a C-terminal CaaX motif by farnesyl- (FPP) and geranylgeranylpyrophosphate (GGPP) is critical for enabling proteins to be inserted into membranes, thus determining their localization and function (McTaggart, 2006). Prenylated proteins include the subunits of trimeric G proteins, protein kinases and more than 150 members of the Ras GTPase superfamily (Takai et al., 2001). These proteins are integral components of complex signaling networks and control diverse cellular activities including intracellular vesicle transport, cell adhesion, endocytosis, cytoskeletal organization, receptor signaling, cell cycle progression and gene expression (Takai et al., 2001).

Recent experimental evidence indicates that isoprenylated GTPases are involved in Alzheimer disease (AD) pathogenesis (Ostrowski et al., 2007; Scheper et al., 2007). Members of the Rab sub-family of small GTPases for example serve as cellular regulators of vesicular transport (Takai et al., 2001). Rab-6 is increased in AD brain and membrane association of this geranylgeranylated protein is dependent on presenilin 1 (PS-1) (Scheper et al., 2004, 2007). PS-1 represents an important physiological facilitator of γ-secretase activity, which promotes the production of the pathologically important amyloid beta protein (Aβ) (Dumanchin et al., 1999; Ridley, 2001; Scheper et al., 2004). It has also been reported that γ-secretase action is stimulated by the isoprenoid GGPP (Urano et al., 2005), for which FPP is the precursor. Both FPP and GGPP were found to increase Aβ levels in H4 neuroglioma cells, expressing the Alzheimer relevant amyloid precursor protein APP695NL (Kukar et al., 2005). Aβ generates oxidative stress, which involves prenylated Rac1 (Lee et al., 2002) and it was suggested that isoprenoids may regulate different Aβ pools in the brain (Cole et al., 2005).

**Fig. 1:**
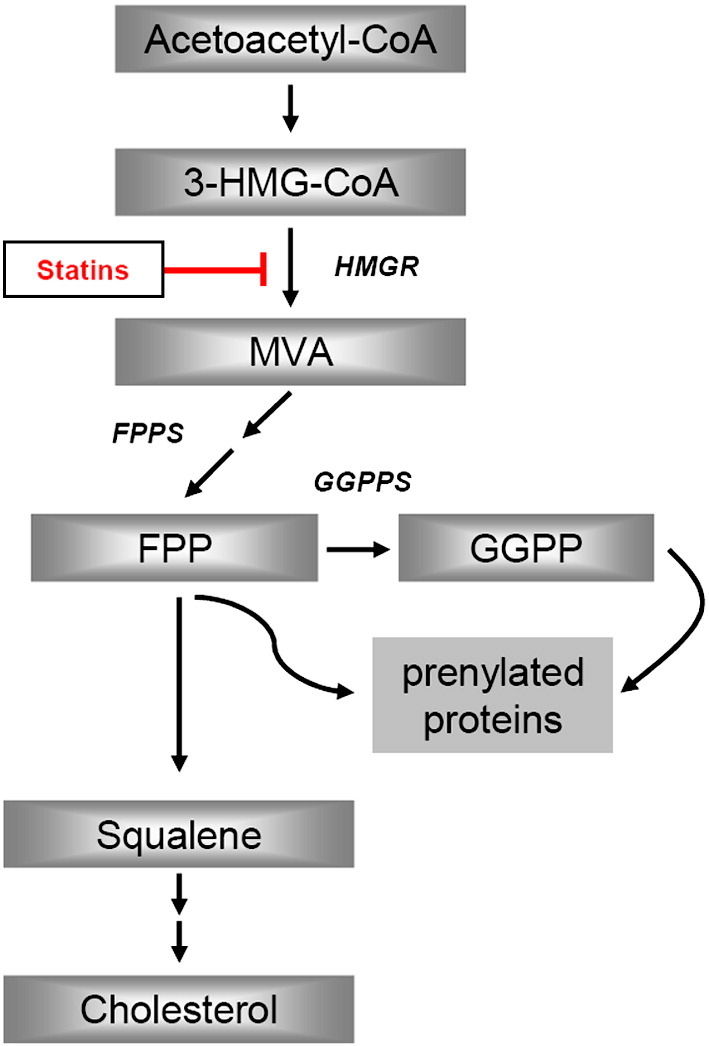
Abbreviated mevalonate/isoprenoid/cholesterol pathway. HMG-CoA-reductase (HMGR) activity leads to mevalonate (MVA), which is a precursor of farnesylpyrophosphate (FPP) formed by FPP synthase (FPPS). FPP is a branching point and serves as a precursor of geranylgeranylpyrophosphate (GGPP) by the activity of GGPP synthase (GGPPS). FPP is also the precursor of cholesterol.

A straightforward prediction derived from the aforementioned studies is that FPP and GGPP levels would be elevated in AD brains as compared with normal neurological controls. However, this hypothesis has not been tested and the absence of such data is in stark contrast to the interest in the role of isoprenoids in protein prenylation and cell function in AD (Cole and Vassar, 2006). Impeding progress on an understanding of FPP and GGPP regulation and consequences on protein targets have been the analytical difficulties of isolation and detection sensitivity of FPP and GGPP (Hooff et al., 2008). We have recently reported FPP and GGPP levels in human brain tissue using a newly developed and validated fluorescence HPLC method (Hooff et al., 2008). In the present study, we determined if FPP and GGPP brain levels and the gene expression of enzymes related to their synthesis are altered in the frontal cortex of AD patients as compared with control samples. FPP and GGPP, like cholesterol, are derived from mevalonate (Fig. 1) and to begin to understand their regulation in brain, effects of inhibition of HMG-CoA reductase on FPP and GGPP levels were determined in mouse brain *in vivo*. It has been previously proposed that brain FPP and GGPP levels are reduced by HMG-CoA reductase inhibition, but neither have such data been reported nor has it been determined if such inhibition has equivalent effects on FPP, GGPP and cholesterol levels.

## Materials and methods

### Chemicals and reagents

FTase and GGTase were obtained from Jena Bioscience (Jena, Germany) and D⁎-GCVLS (dansyl gly-cys-val-leu-ser) and D⁎-GCVLL (dansyl gly-cys-val-leu-leu) from Calbiochem (Darmstadt, Germany). Ammonium hydroxide solution 28–30% was purchased from Alfa Aesar (Karlsruhe, Germany), the phosphatase inhibitors Halt^®^ and Phosstop^®^ from Thermo-Fisher/Piercenet (Bonn, Germany) and Roche Diagnostics GmbH (Mannheim, Germany), respectively. All solvents were of analytical grade or higher quality. Acetonitrile was obtained from Carl Roth GmbH (Karlsruhe, Germany), 1-butanol, n-hexane, 2-propanol, methanol, acetone, ammonium acetate and assay buffer compounds: Tris–HCl, MgCl_2_, ZnCl_2_ and Na_2_CO_3_ were obtained from Merck (Darmstadt, Germany). FPP, GGPP, octyl-β-d-glucopyranoside and dithiothreitol were from Sigma-Aldrich (Schnelldorf, Germany). Millipore water was used for all solutions (Schwalbach, Germany).

### Human brain samples

Postmortem tissue from the frontal cortex was obtained from the Human Brain and Spinal Fluid Resource Center, VA West Los Angeles Healthcare Center, 11301 Wilshire Blvd. Los Angeles, CA 90073 which is sponsored by NIH-NINDS/NIMH, National Multiple Sclerosis Society and the Department of Veterans Affairs. In this initial study of FPP and GGPP levels, brain grey and white matter of the cerebral cortex was examined. Recent studies in AD patients indicate changes in grey matter associated with hypometabolism, specific neuropsychiatric symptoms and behavior (Bruen et al., 2008; Chetelat et al., 2008). However, to confirm the specificity of the isoprenoid elevation, we additionally measured FPP, GGPP, and cholesterol levels in the white matter of the same samples. At autopsy, brains were cut into coronal sections (2–4) and a sample of frontal cortex tissue of each sample was frozen and stored at − 80 °C. Patients with AD were classified according to the criteria of the Consortium to Establish a Registry for AD (CERAD) and Braak stages. Controls were neurological normal. The donor age varied between 70 and 88 years and the average post mortem interval (PMI) was 15.5 h (see Table 1). No correlation between individual PMI and FPP or GGPP levels were found (data not shown). For sample preparation, a small piece of frozen cortex was immediately dissected into grey and white matter and homogenized.

### Western blot analysis

For NeuN protein determination, samples were prepared by diluting 15 μg protein with the reducing agent (10×) and loading buffer (4×). After denaturation for 10 min at 95 °C, the samples were electrophoretically separated on a 4–12% NuPage Bis/Tris gel (Invitrogen, Germany) for 40 min at 180 V and then transferred on a PVDF membrane for 120 min at 30 V and incubated with the primary antibodies anti-NeuN (MAB377, Millipore, Germany), anti-GAPDH (MAB374, Millipore, Germany), followed by secondary antibodies (Calbiochem, Germany) conjugated to horseradish peroxidase and processed for visualization by ECL® reagent (Amersham Bioscience). Band analysis was performed using Bio-Rad's Quantity One Software.

### Tissue preparation and HPLC analysis

Brain tissue preparation and FPP and GGPP determination have previously been described by our group (Hooff et al., 2008). Briefly, each brain tissue sample was homogenized with a rotor-stator homogenizer at 1100 rpm in 100 mM Tris buffer (pH 8.5) containing 5 μL Halt® and 10 μL Phosstop® phosphatase inhibitors. The homogenate was vigorously mixed with 1 mL 100 mM Tris buffer (pH 8.5). A 50 μL aliquot from the homogenate was retained for protein and cholesterol determination and then spiked with 15 μL 2.8 μM solution of 5-(dimethylamino)naphthalene-1-(4-nonylphenol)-sulfonic acid ester used as the internal standard (IS). The mixture was loaded onto Merck Extrelut® NT1-columns (Darmstadt, Germany) and after 15 min eluted with a total of 6 mL of a 1-butanol–ammonium hydroxide–water mixture (10:1:2, v/v/v). The filtrate was centrifuged for 10 min at 29,000 ×*g* to remove precipitated proteins. The supernatant was evaporated under reduced pressure and dissolved again in 5 mL 5% methanol. After sonication, the solution was applied to Oasis® HLB (3 cc; 60 mg) solid-phase extraction cartridges from Waters (Eschborn, Germany) previously conditioned with n-hexane, 2-propanol and methanol. The extract was washed with a 2% methanol solution and finally eluted with an ammonium hydroxide–propanol–n-hexane mixture (1:7:12, v/v/v). The filtrate was vacuum-dried to be re-dissolved in assay buffer for the enzymatic reaction. The pre-column enzymatic attachment of dansyl-labeled pentapeptides to FPP and GGPP allows a highly sensitive HPLC fluorescence detection (HPLC-FLD).

For pre-column dansyl-labeling, the dried residue was dissolved in 44 μL Tris–HCl assay buffer (Tong et al., 2005) and spiked with 2 μL of a 50 μM solution of D⁎-GCVLS and D⁎-GCVLL (dansyl-labeled peptides) and 250 ng FTase and GGTase, respectively. The mixture was incubated at 37 °C in an Eppendorf thermomixer comfort (Wesseling-Berzdorf, Germany) programmed for 90 min (per minute: 5 s; 500 rpm). After stopping the reaction, the mixture was centrifuged (4 °C; 15,000 ×*g*; 5 min) prior to HPLC-FLD analysis. The chromatographic separation was carried out on a Jasco HPLC-system (LG-980-02, PU-980, AS-950; Gross-Umstadt, Germany) with a gradient elution on an Ascentis® Express C-18 reversed-phase analytical column from Supelco (150 × 2.1 mm, 2.7 μm; Munich, Germany) protected by a Phenomenex Security guard column (C-18, 4 × 2.0 mm; Aschaffenburg, Germany). Two solvents were used for gradient elution: solvent A, 20 mM ammonium acetate in 40% acetonitrile and solvent B, 20 mM ammonium acetate in 90% acetonitrile. The gradient was initiated at 35% solvent B for 1.5 min, subsequently ramped linearly to 100% within 6.5 min, held for 6 min and then brought back to 35% solvent B within 2 min. The total run time was 20 min with a constant flow rate of 0.5 mL/min at 20 °C. The labeled analytes were monitored by a fluorescence detector (Gilson, Middleton, USA) set at an excitation wavelength of 340 nm and 525 nm for emission. The retention times for the labeled FPP and GGPP were 4.1 min and 11.0 min, respectively and 12.1 min for the internal standard.

### Simvastatin administration to mice

Simvastatin was administered to mice as previously reported (Johnson-Anuna et al., 2005). C57BL/6J mice (16 to 21 g) were obtained from Janvier, Le Genest, France. Mice were maintained on a 12-h dark–light cycle with pelleted food and tap water *ad libitum*. All experiments were carried out according to the European Communities Council Directive (86/609/EEC) by individuals with appropriate training and experience. Statin suspension was prepared daily in 0.2% (w/v) aqueous agarose gel giving a final concentration of 10 mg simvastatin/mL. Animals received 50 mg simvastatin/kg b.w. daily for 21 days or vehicle by oral gavage (diameter: 1 mm) and a maximal application volume of 0.5 mL. This treatment regime was chosen, since according to our previous work a significant reduction of brain cholesterol levels without any toxicological effects, e.g. apoptosis, was observed (Johnson-Anuna et al., 2005; Franke et al., 2007). At the end of the study, animals were euthanized by cervical displacement 2 h after the last drug treatment. The cerebrum was removed, snap frozen in liquid nitrogen, and stored at − 80 °C prior to isolation and analysis. For the isolation, half of a hemisphere was treated as described in the preparation procedures of the human brain samples and analyzed accordingly.

### Protein and cholesterol assays

Protein concentrations were measured using the BCA Protein Assay Kit from Thermo-Fisher/Pierce (Bonn, Germany). Samples were measured in triplicates. Total cholesterol levels were determined enzymatically, using the CHOD-PAP method (Roche Diagnostics GmbH, Mannheim, Germany).

### Quantitative real-time polymerase chain reaction (qRT-PCR)

#### RNA isolation

Brain tissue samples were homogenized in 1 ml of Invitrogen's TRIzol® reagent. The remaining isolation procedure was performed in accordance to the manufacturer's instructions. The resulting RNA was re-suspended in nuclease-free water.

#### Reverse transcription

RNA concentration was determined by measuring the absorbance at 260 nm. Known amounts of RNA were then reverse-transcribed using Bio-Rad's iScript™ cDNA synthesis kit according to manufacturer's instructions, which resulted in an equivalent amount of cDNA.

#### Primer design

Internal primers were designed for each of the targets in question. The NCBI's Entrez Gene database was the source of the nucleotide sequences of each gene. Using the BLAST program, intron/exon borders were identified and primers were designed to amplify around these regions to eliminate amplification of genomic DNA rather than the desired cDNA. Primer sequences were generated using MIT's program “Primer3”. Primer pairs are as follows:

GAPDH: left 5′- GAA ATC CCA TCA CCA TCT TCC -3′
right 5′- ATG GTT CAC ACC CAT GAC G -3′
200 base pair product
HMGR: left 5′- GAG GCA TTT GAC AGC ACT AGC -3′
right 5′- TGC ATT TCA GGG AAA TAC TCG -3′
179 base pair product
FPPS: left 5′- GAA GAT CCT GCT GGA GAT GG -3′
right 5′- GTT GTC CTG GAT GTC AGT GC -3′
109 base pair product
GGPPS: left 5′- CTA TCT GGG CAG TTC CAA GC -3′
right 5′- GAC TTC AGT GAG GGT GTT TCC -3′
127 base pair product.

Primers were tested in a RT-PCR experiment using Invitrogen's SuperScript™ III One-Step RT-PCR System. The reaction products were run on an agarose gel containing ethidium bromide and imaged under UV light to verify that the intended targets were being amplified.

#### Real-time quantitative PCR

Relative abundance of mRNA was measured through real-time analysis using Invitrogen's SYBR® GreenER™ qPCR SuperMix. Thermal cycles were programmed according to the manufacturer's instructions with annealing temperatures of 56.8 °C, 57.0 °C, 56.5 °C, and 58.5 °C for HMGR, FPPS, and GGPPS primers respectively. In each 50μl reaction, there were 100 ng of cDNA template and a 200 nM concentration of left and right primers. GAPDH was used as a reference gene to normalize the data for each experiment and therefore gene fold expression was determined using ΔΔC_T_ calculations.

### Statistics

All data are expressed as mean values ± standard error of the mean (SEM) unless stated otherwise. For direct comparison of differences between two groups, students' *t*-test was calculated. All calculations were performed with GraphPad Prism version 5.00 for Mac, GraphPad software, San Diego, USA.

## Results

**Fig. 2:**
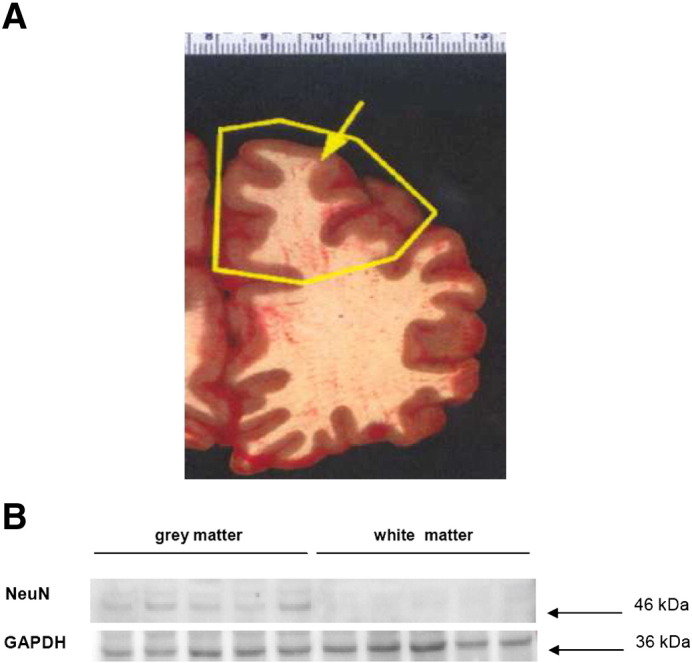
Preparation of brain tissue from control and AD samples. (A) At autopsy, a picture was taken of each sample showing the excision area and a coronal section is indicated. (B) Quality of separation of grey from white matter was confirmed by enrichment of the protein NeuN, which is a marker of neuronal cell bodies. Protein abundance was determined by Western analysis as described in the Materials and methods section. Five samples of each tissue section were used for the protein analysis.

The isoprenoids FPP and GGPP were determined simultaneously using HPLC-FLD in the frontal cortex of male AD patients and age-related controls. Brain grey and white matter of the cerebral cortex was examined in this initial study of FPP and GGPP levels. Changes in grey matter of AD patient have been reported recently to be associated with hypometabolism, specific neuropsychiatric symptoms and behavior (Bruen et al., 2008; Chételat et al., 2008). Purity of both, the grey and white matter preparation was confirmed by enrichment of the neuronal cell nucleus marker protein NeuN (Fig. 2).

**Fig. 3:**
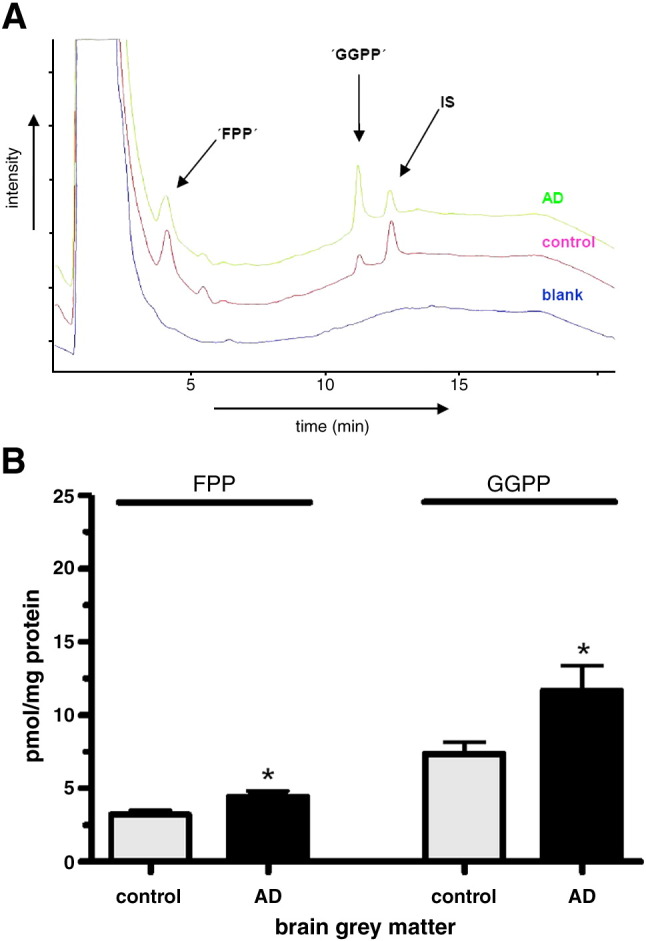
Elevated FPP and GGPP levels in the frontal cortex grey matter of male AD brain compared with controls. (A) Representative chromatographic overlay of grey matter brain samples. Upper trace: AD sample; middle trace: control sample; lower trace: blank sample. Peak labeling: ‘FPP’ and ‘GGPP’ stand for the respective dansyl-labeled isoprenoids. (B) Endogenous FPP and GGPP levels in human brain grey matter in the control and AD group. Results are shown as means ± SEM, ⁎*p* < 0.05; *n* = 13 for each group.

This study shows for the first time FPP and GGPP levels in brain tissue of AD patients and normal neurologic controls. FPP and GGPP levels were determined by HPLC-FLD and representative chromatograms are shown in Fig. 3A. It can be seen in Fig. 3B that GGPP levels were significantly higher in brain tissue of AD patients (56%) as compared with control samples. FPP levels were also significantly higher in the AD brain tissue 36% (Fig. 3B). In both AD patients and controls, GGPP levels were markedly higher than FPP levels and that finding is consistent with two recent reports in mouse brain and normal human brain (Hooff et al., 2008; Tong et al., 2008).

**Fig. 4:**
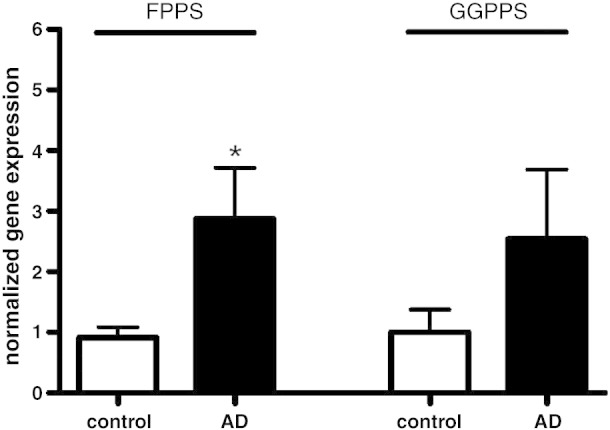
Gene expression of the FPP and GGPP synthase in human AD and control brain samples. Gene expression was determined by qRT-PCR as described in the Materials and methods section. Data were normalized to GAPDH and results are shown as means ± SEM; ⁎*p* < 0.05; *n* = 10 for each group.

FPP synthase and GGPP synthase are two genes whose protein products synthesize FPP and GGPP. Gene expression of FPP synthase and GGPP synthase were determined in brain tissue of the AD and control samples by qRT-PCR. The increase in FPP levels in the frontal cortex of AD brain was associated with a significant up-regulation of FPP synthase gene expression as seen in Fig. 4. GGPPS mRNA levels were increased but differences did not reach significance (Fig. 4). FPP and GGPP levels may be controlled by enhanced specific activity of the two synthases rather than by enhanced production of mevalonate due to up-regulation of HMGR.

**Fig. 5:**
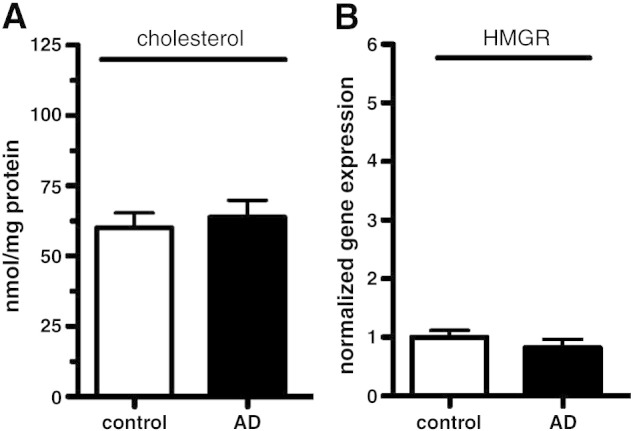
Cholesterol levels and HMG-CoA reductase gene expression in brain tissue of AD and control subjects. (A) Cholesterol levels in brain tissue of control and AD samples; *n* = 13 for each group. (B) Gene expression of HMG-CoA reductase (HMGR) in brain tissue of controls and AD samples. Gene expression was determined in grey matter by qRT-PCR as described in the Materials and methods section. Data were normalized to GAPDH and results are shown as means ± SEM; *n* = 10 for each group.

**Fig. 6:**
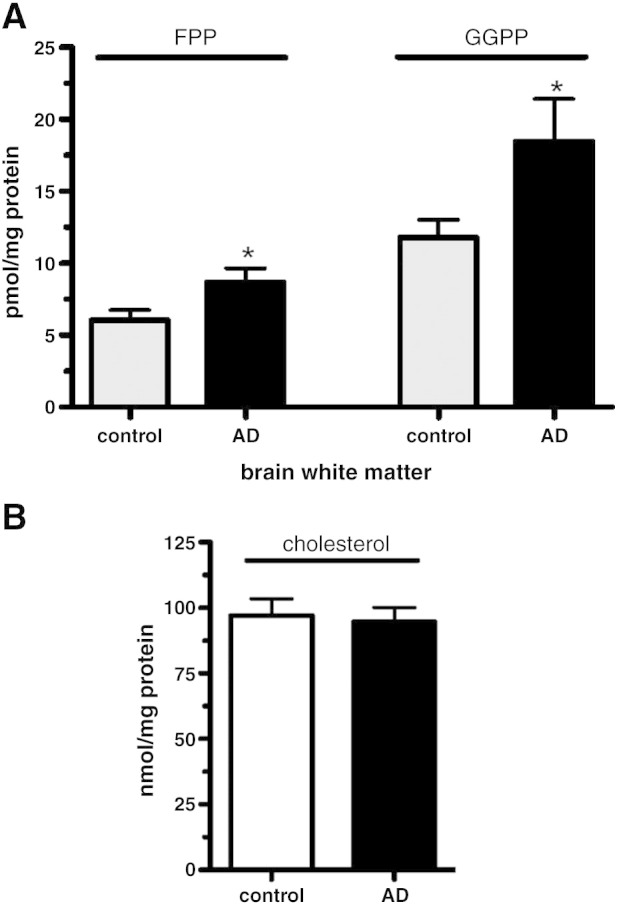
Elevated FPP and GGPP levels in the frontal cortex white matter of male AD brain compared with controls. (A) Endogenous FPP and GGPP levels in human brain white matter (B) Cholesterol levels in brain tissue of control and AD samples; *n* = 13 for each group. Results are shown as means ± SEM, ⁎*p* < 0.05; *n* = 13 for each group.

FPP serves as a precursor of both GGPP and cholesterol (Fig. 1). Our data show significantly increased levels of FPP and GGPP in brain tissue of AD patients as shown in Fig. 3B. On the other hand as noted in Fig. 5, cholesterol levels and gene expression of HMG-CoA reductase were similar in AD and control samples. Taken together these results indicate that homeostasis of FPP and GGPP but not cholesterol is specifically targeted in brain tissue of AD patients. The specificity of the isoprenoid elevation is further supported by similar observations in white matter brain tissue (Fig. 6A). Here the same AD brains show a 59% and 43% elevation for GGPP and FPP, respectively. Again this observation is not accompanied by an alteration of the cholesterol concentration (Fig. 6B).

**Fig. 7:**
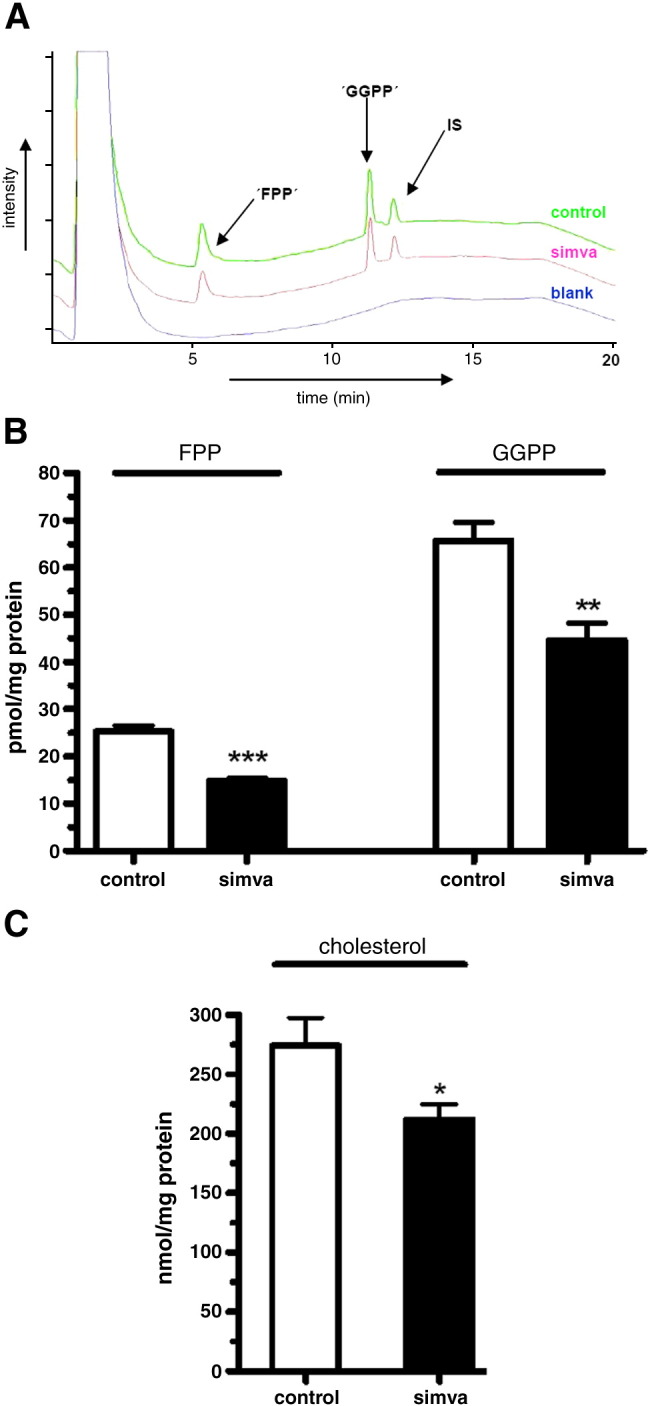
Brain FPP, GGPP and cholesterol levels in mice treated with the HMG-CoA reductase inhibitor simvastatin. (A) Representative chromatographic overlay of mouse brain samples. Upper trace: control, middle trace: simvastatin treated sample and lower trace: blank sample. (B) FPP and GGPP levels in the cerebrum of control and simvastatin treated mice. (C) Brain cholesterol levels in control and simvastatin treated mice. Results are shown as means ± SEM; ⁎⁎⁎*p* < 0.001, ⁎*p* < 0.01 and⁎*p* < 0.05; *n* = 6 for each group.

To establish if FPP and GGPP regulation is more susceptible to perturbation than cholesterol, we determined FPP, GGPP and cholesterol levels in brains of mice treated with the HMG-CoA reductase inhibitor, simvastatin. It has been previously proposed that statin treatment would reduce brain FPP and GGPP levels (Cole et al., 2005), but such data have not been reported. FPP and GGPP levels were determined by HPLC-FLD and representative chromatograms are shown in Fig. 7A. Data in Fig. 7B show that inhibition of HMG-CoA reductase significantly reduced FPP and GGPP by 52% and 33%, respectively. Cholesterol levels were also significantly reduced but to a lower extent (22%) as shown in Fig. 7C. In AD brain, FPP and GGPP levels are elevated (Fig. 3B) but cholesterol levels and HMG-CoA reductase gene expression remain unaffected (Fig. 5A). In contrast, perturbation of HMG-CoA reductase alters both isoprenoids and cholestrol levels, albeit with differences in magnitude (Figs. 7B, C). Regulation of FPP and GGPP would appear to be altered in AD brain which may not involve HMG-CoA reductase.

## Discussion

The mevalonate/isoprenoid/cholesterol pathway has generated much attention in AD (Cole and Vassar, 2006; Scheper et al., 2007). Of particular interest has been the proposed involvement of the two isoprenoids FPP and GGPP via their function in prenylating small GTPases. One of the protective effects of statins in brain has been attributed to a reduction in FPP and GGPP levels (Cole and Vassar, 2006; McTaggart, 2006). In spite of the keen interest in isoprenoids and AD, evidence supporting involvement of isoprenoids has only been indirect due to a lack of a quantitative analytical method for determining FPP and GGPP levels. This study provides for the first time data indicating that homeostasis of FPP and GGPP but not cholesterol is specifically targeted in brain tissue of male AD patients. Another important finding of this study is that we show for the first time effects of HMG-CoA reducatse inhibition on FPP and GGPP levels in brains of mice chronically treated with simvastatin.

Results of this study suggest that isoprenoid regulation may be specifically altered in AD. This conclusion is based on the following. Both FPP and GGPP levels were significantly higher in brain tissue of male AD patients as compared with male control samples. Brain cholesterol levels on the other hand did not differ between the two groups. HMG-CoA reductase gene expression was similar between the AD and control groups. However, when HMG-CoA reductase was inhibited, FPP, GGPP and cholesterol levels were significantly reduced in brain tissue of mice chronically treated with simvastatin. Finally, gene expression of FPPS was significantly elevated in AD brain samples compared to control brain samples. The up-regulation of FPPS is a key finding because it is a precursor of both GGPP and cholesterol (Fig. 1).

Regulation of FPP and GGPP is not as well-understood as cholesterol regulation and this especially applies to the brain. Low cellular cholesterol levels are sensed by the sterol regulatory element-binding protein-cleavage activating protein (SCAP), which in turn triggers a cascade of events ultimately leading to the up-regulation of the HMG-CoA reductase and increased mevalonate production (reviewed in Hampton, 2002). Mevalonate is the upstream precursor of several products including FPP. Synthesis of FPP involves the enzyme FPPS and condensation reactions of isopentenyl-5-PP and geranyl-PP. As shown in the present study, both FPP levels and FPPS gene expression were elevated in male AD brains. What has not been clearly established is if changes in FPP levels are detected by SCAP leading to the activation of the sterol regulatory element-binding protein-2 (SREBP-2). In CaCo-2 colon epithelial cells, addition of exogenous FPP did not alter mature SREBP-2 abundance in mevalonate depleted cells by lovastatin whereas adding back mevalonate counteracted effects of lovastatin on mature SREBP-2 (Murthy et al., 2005). Based on elevated FPP levels and FPPS gene expression in contrast to unchanged cholesterol levels and HMG-CoA reductase gene expression, a tentative conclusion is that the increase in FPP levels in AD brain may not involve the SCAP/SREBP-2 pathway.

GGPP levels but not cholesterol levels were significantly higher in AD brain. FPP serves as a precursor of both GGPP and cholesterol. One interpretation of the data is a possible shunting of FPP to GGPP and away from cholesterol. While gene expression of GGPP synthase was higher in AD samples than controls it did not reach significance. It is possible in the case of GGPP that enzymatic activity of GGPP synthase may be a critical regulatory contributor to its synthesis. The stimulus for the increase in GGPP as well as FPP levels in AD brain tissue is unclear. A speculative explanation may reside in proteins that are prenylated by the two isoprenoids. It was reported that both FPP and GGPP levels were increased when reticulocyte cytosol was incubated with recombinant protein substrates for FPP and GGPP prenylation, respectively (Lutz et al., 1992).

GGPP levels were higher than FPP levels in both the male AD and control brain samples and mouse brain samples. Those results are consistent with the recent reports on FPP and GGPP levels in mouse brain (Tong et al., 2008) and normal human brain tissue (Hooff et al., 2008). An explanation for such differences is not well-understood in any tissue and calls attention to the need for future studies. There is some evidence that more proteins are prenylated by GGPP than FPP (Roskoski, 2003) but at the same time the utilization of FPP is much greater than GGPP owing to it serving as a precursor for cholesterol, GGPP and longer chain isoprenoids.

For the first time, effects of inhibition of HMG-CoA reductase by a statin on brain FPP and GGPP levels were observed. It has been previously proposed (Cole and Vassar, 2006) that HMG-CoA reductase inhibition would reduce brain FPP and GGPP levels but such data had not been reported due to analytical difficulties of isolation and detection sensitivity of FPP and GGPP (Hooff et al., 2008). We show in mouse brain that simvastatin had a greater effect on reducing FPP levels followed by reductions in GGPP and cholesterol levels. It would appear that HMG-CoA reductase inhibition does not have an equal effect on the downstream products of mevalonate. The consequences of these differential effects on isoprenoids and cholesterol on cell function are unclear. One possibility is that there is a gradient of cellular effects based on the magnitude of reduction in the individual lipids with FPP associated cell function more affected.

In summary, the results of the current study show that FPP and GGPP levels in grey and white matter were elevated in male AD brain tissue. While further work is needed to unequivocally demonstrate that FPP and GGPP regulation is altered in AD including studies examining brain tissue of other neurodegenerative diseases, the present findings suggest that abundance of prenylated small GTPases may be increased in AD (Cole and Vassar, 2006; Scheper et al., 2007). Also, these findings may have utility in the development of new drugs similar to bisphosphonates which inhibit FPP synthase but that could pass the blood brain-barrier. Finally, the specific increase in isoprenoids in the male AD brain but not cholesterol may help to explain the varying clinical effects of statins on treating AD (Li et al., 2007; Sparks et al., 2008; Zhou et al., 2007).

## Figures and Tables

**Table 1:** Patient information on control and Alzheimer subjects.

	Control (*n* = 13)	Alzheimer (*n* = 13)	*P* value
Demographic			
Age at death (years)	77.8 ± 3.5	80.4 ± 4.8	0.133
Gender	13 male	13 male	
Histological			
Postmortem interval (h)	15.0 ± 4.4	16.5 ± 5.0	0.433
Brain hemisphere (left:right)	0:13	3:10	
Braak — Stage (I–V)	–	5.1 ± 0.7	

At autopsy, brains were cut into coronal sections (2–4) and a piece of frontal cortex tissue of each sample was freshly frozen and stored at − 80 °C. Patients were classified as AD or control cases according to CERAD. Results are shown as means ± SD.
